# Computational and experimental analysis identifies Arabidopsis genes specifically expressed during early seed development

**DOI:** 10.1186/1471-2164-7-38

**Published:** 2006-02-28

**Authors:** Cristian Becerra, Pere Puigdomenech, Carlos M Vicient

**Affiliations:** 1 Laboratori de Genetica Molecular i Vegetal, CSIC-IRTA, Jordi Girona 18–36, 08034, Barcelona, Spain

## Abstract

**Background:**

Plant seeds are complex organs in which maternal tissues, embryo and endosperm, follow distinct but coordinated developmental programs. Some morphogenetic and metabolic processes are exclusively associated with seed development. The goal of this study was to explore the feasibility of incorporating the available online bioinformatics databases to discover Arabidopsis genes specifically expressed in certain organs, in our case immature seeds.

**Results:**

A total of 11,032 EST sequences obtained from isolated immature seeds were used as the initial dataset (178 of them newly described here). A pilot study was performed using EST virtual subtraction followed by microarray data analysis, using the Genevestigator tool. These techniques led to the identification of 49 immature seed-specific genes. The findings were validated by RT-PCR analysis and *in situ *hybridization.

**Conclusion:**

We conclude that the combined *in silico *data analysis is an effective data mining strategy for the identification of tissue-specific gene expression.

## Background

Seeds are complex genetic entities with a diploid maternal genotype, derived from the ovary wall, a diploid embryo, with equal genetic contributions from the pollen donor and pollen recipient, and a triploid endosperm, in which the maternal genetic contribution is twice that of the paternal parent. Endosperm development is a process with many unique features determining the coordinated development and disappearance of a highly specialized organ [1]. During embryogenesis, the egg cell divides and develops into an embryo, passing through different developmental phases: globular, heart, torpedo, cotyledon, curled-cotyledon and maturation [2]. Key steps in early embryo development are the acquisition of a polar structure with a shoot-root axis, the formation of the apical and root meristems, and the differentiation of the cotyledon primordia. After this last stage, the size of the embryo increases and deposition of storage macromolecules begins. Finally, during maturation, the embryo desiccates. During this process, the seed coat develops from the two integuments that surround the embryo. Several of the processes described above are not present in any other plant tissues, so the genetic program for seed development is likely to involve the concerted activity of many seed-specific genes.

Determination of the genes involved in seed development, and their functions, is one of the major goals in plant developmental biology. Mutational approaches have been extensively used to analyse seed development in Arabidopsis [3-5]. Several mutants have been isolated giving loss-of- or altered-seed development allowing the identification of several genes [6,7]. However, insertional mutagenesis has some deficiencies. For example, probably due to gene redundancy, many of the insertions in genes do not produce any detectable phenotype, and genes whose disruption produces alterations in seed development are not necessarily genes with seed specific expression [6]. In consequence, although mutational approaches have been, and still are, basic for understanding the processes involved in seed development, they are not enough to build a complete picture of the process.

Expression profiling and definition of genes specifically or preferentially expressed in certain tissues complement the genetic and molecular approaches. The generation of EST collections and the oligonucleotide-based microarrays can produce reliable, high-quality data [8,9]. The deposition of the results of RNA profiling experiments in public databases provides a valuable tool for *in silico *analysis of organ specific gene expression. There have been several reports of EST-based computer analysis of human tissue transcriptomes [10-15], and computer analyses have been performed in differential human EST database searches [16].

EST abundance in plants is not as high as for humans, but for some species the total number of ESTs in publicly available databases exceeds the total number of genes by more than one order of magnitude. For example, the NCBI dbEST database release 111105 (November 11, 2005) [17] included 656,945 from *Zea mays *(maize), 600,039 sequences from *Triticum aestivum *(wheat), 420,789 from *Arabidopsis thaliana *(thale cress) and 406,790 from *Oryza sativa *(rice), compared with the 7,057,754 for humans. Despite this, there are few examples of *in silico *expression studies in plants [18,19].

From the complete sequencing of certain plant genomes, it is possible to monitor gene expression on a genome-scale using high-density oligonucleotide arrays [20]. Thousands of Arabidopsis arrays, containing probes for more than twenty thousand genes, have been processed, and systematic analyses of gene expression in different organs, developmental conditions and stress responses, have been performed [9,21-23]. The results of many of these are publicly available through web browser interfaces such as the Genevestigator tool [24-26]. In view of this, at least for Arabidopsis, data analysis rather than data collection is the first challenge for biologists in determining patterns of gene expression.

The focus of this work was the identification of genes whose expression is specific in immature seeds. Firstly, we sequenced cDNA clones from isolated immature seeds. Secondly, we used *in silico *subtraction in a combination of EST selection and microarray data analysis in order to select genes with the desired pattern of expression. Finally, 49 genes specifically expressed during seed development were selected. Our study demonstrates the reliability of *in silico *subtraction methods in Arabidopsis and provides a basis for targeted reverse-genetic approaches aimed at identifying key genes involved in reproductive development in plants.

## Results and discussion

### Sequencing Arabidopsis young seed ESTs

**Figure 1:**
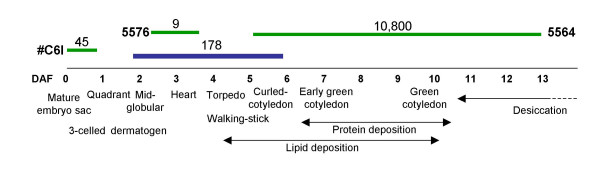
**Overview of EST libraries from isolated immature Arabidopsis seeds. **At the top, a representation of the available EST collections extracted from immature seeds. Lines in colour represent the period of development covered by the library. The library code according to the TIGR Arabidopsis Gene Index ( [29]) is indicated next to the line. The number of ESTs available from the corresponding library is indicated above the line. Green lines correspond to previously existing EST collections, and the blue line corresponds to the new library described here. At the bottom, the stages of embryo and seed development, related to days after flowering (DAF), is shown [49]. The main processes associated with seed development are indicated.

ESTs from isolated Arabidopsis immature seeds are not very abundant in EST databases (Figure 1). Among the 420,789 Arabidopsis ESTs deposited (release 111105) [17], 10,854 correspond to isolated immature seeds, 10,800 correspond to seeds in mid-development stages [27] and only 54 were obtained from early stages of seed development. We constructed a cDNA library from developing Arabidopsis seeds isolated at a stage from mid-globular to curled-cotyledon (2 to 6 days after pollination) and obtained 178 single pass 5' end sequences (>140 bp). The average sequence length was 579 bp. Newly sequenced ESTs were assembled in contigs and gene identities were assigned querying against the Arabidopsis genome database at TAIR [28] using the BLAST algorithm. They corresponded to 95 individual genes: 93 nuclear and two from chloroplasts. Functional categories were determined based on GO data in the TAIR database [28]. 21% of the genes are linked to translation, 6% to carbohydrate metabolism and 5% to development. The function of 31% of the genes remained unknown. For two of the genes (At1g60987 and At2g02490) no ESTs have been previously sequenced.

### Identification of genes specifically expressed in seeds during early development

A two step *in silico *subtraction procedure was used to select genes specifically transcribed in immature seeds. The first selection step was based on EST abundance and the second step on microarray data analysis.

The objective of the first step was to identify genes having ESTs only from immature seeds and not from other organs. We divided the Arabidopsis EST libraries deposited in the TIGR Arabidopsis Gene Index [29] into three categories, according to the organs they were made from (Additional file 1):

a) Immature seed: this includes 10,854 ESTs from four cDNA libraries (Figure 1).

b) Other tissues: this includes 50,992 ESTs from 78 cDNA libraries obtained from vegetative tissues, non-pollinated flowers and dry seeds.

c) Non-informative: this includes libraries obtained from mixed organs and whole plants, including libraries from siliques.

Subtraction was done based on the EST contigs and gene assignations in TIGR Arabidopsis Gene Index [29]. We selected genes having corresponding EST sequences in category a (immature seeds) and not in category b (other tissues). 640 genes passed our first subtraction criteria (Additional file 2). Two correspond to chloroplast genes, three to mitochondrial genes and 26 had homology to parts of the Arabidopsis genome in which no genes have been reported.

The second selection step was based on the Arabidopsis Affymetrix GeneChip^® ^average data available on the Genevestigator analysis tool site [24-26]. We used the meta-analyzer program, which performs a heat map of normalized signal intensity values, corresponding to the different organs of the plant, for each gene. Values range from 0 to 100, 100 being the highest level of expression. We selected the genes using the following criteria:

(i) The expression in seeds should be higher than 80.

(ii) The expression in other organs should be lower than 5, except for siliques, carpels and inflorescences, as these three organs could contain immature seeds at the very early stages after pollination. Detected level 5 is probably low, but was chosen in order to avoid possible errors in the normalisation algorithm in the meta-analyzer program.

(iii) The expression level in seeds should be higher or equal to the expression in siliques, carpels or inflorescences.

49 of the 634 selected genes were not considered in the second analysis because they are not included in the Arabidopsis Affymetrix 22K GeneChip^®^. Of the remaining 585 genes, 49 (8%) fulfilled the selection criteria and may represent genes specifically expressed in immature seeds (Table 1). From the non-selected genes, 51% did not fit the selection condition (i), 96% the selection condition (ii) and 35% the selection condition (iii). Surprisingly, 21% of the genes showed higher values in siliques than in seeds. The different conditions in which tissues were collected for cDNA synthesis and microarray hybridizations could explain these results.

The advantage of the selection method is demonstrated by the presence of several genes already characterized as specifically expressed in seeds, such as: *abi3 *[30]; At1g48130, encoding a peroxiredoxin (PER1) whose expression is restricted to seeds [31]; At1g67100, which is homologous to the *Brassica Bn15D17A *gene, highly and specifically expressed in embryos and seed coat at the early stages of seed development [32]; and At5g07190 and At5g55240, which encode embryo-specific proteins isolated in the course of a differential display experiment [33].

We also tested the direct application of the microarray subtraction without EST selection. We chose the first 1,500 genes from chromosome 1 (according to the AGI code) included in the Arabidopsis Affymetrix 22K GeneChip^® ^(from At1g01010 to At1g18340). 28 of the 1,500 genes (1.9%) fell within the microarray-based selection criteria. If there is the same proportion in the whole genome, about 550 genes would be selected. These results indicate that Genevestigator may be a useful tool to investigate organ specific gene expression in Arabidopsis. However, data obtained from Genevestigator is based on the normalised average signal intensity values obtained from several array experiments [24-26]. The normalisation algorithms used to generate Genevestigator values could introduce false positives and negatives, particularly for genes with low levels of expression. In consequence, combining Genevestigator results with EST abundance data gives a more reliable dataset of genes specifically expressed in a certain organ, seeds in our case.

### Experimental validation of the patterns of expression of the selected genes

**Figure 2:**
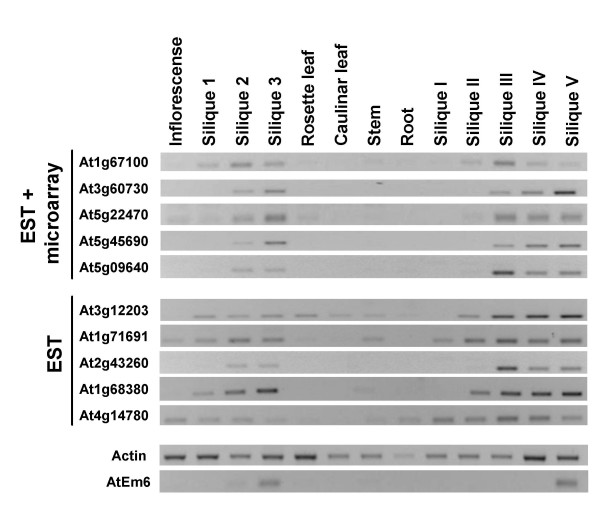
**RT-PCR analysis of the expression profiles of ten genes isolated by in silico screening. **"EST + microarray" indicates genes isolated by the combination of EST selection and microarray data analyses. "EST" indicates genes isolated only by EST selection. Siliques 1 to 3 correspond to whole siliques at different stages of development (1, young green; 2, green fully developed; 3, desiccating siliques). Siliques I to V correspond to siliques at different stages of development (I, 0–4 daf; II, 4–8 daf; III, 8–12 daf; IV, 12–16 daf; V, 17–21 daf). In each case, the size of the bands was as expected.

We used RT-PCR to check our selection procedure (Figure 2). Ten genes were selected, five of which were only used in the EST based selection and not the microarray, and the other five genes passed both selection steps. Two genes were used as additional controls: actin, which is expressed in all tissues, and *AtEm6*, which is specifically expressed during late embryogenesis [34]. All 10 genes analyzed showed higher expression levels in siliques, but silique specificity is, in general, higher in the genes selected by EST and microarray than in the genes selected only by EST subtraction. Two of the genes in the EST and microarray group, At1g67100 and At5g22470, gave low levels of amplification in rosette leaves and At1g67100 also in stem. This difference between Genevestigator and experimental data could be a consequence of different levels of detection in RT-PCR and microarray experiments or different experimental conditions. They do not indicate strong bias in the results. EST and microarray based selection produces a specific, expression-based, list of genes.

**Figure 3:**
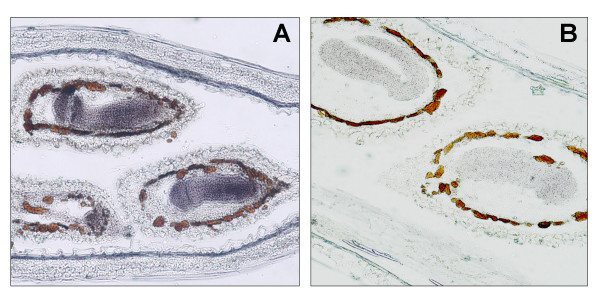
***In-situ *hybridization analysis of a seed-specifically expressed gene. **Seed-specific transcript labelling of embryos at the late torpedo stage as shown by in situ hybridization of transverse sections of Arabidopsis siliques probed with digoxigenin-labelled *At5g22470 *mRNA, viewed under bright-field optics.

Seed specific expression was further demonstrated by *in situ *hybridization for the At5g22470 gene encoding a Poly (ADP-ribose) polymerase family protein (PARP) (Figure 3). The At5g22470 transcripts were detected specifically in the embryo and not in the endosperm, pericarp, valves or septum. The profile of the expression of the At5g22470 gene is consistent with the predicted seed specific transcription.

The RT-PCR experiments and the presence of genes known to be specifically expressed in seed demonstrate that the selection procedure identifies genes specifically, or at least, predominantly, expressed in developing seeds. The relatively low number of genes selected is probably a consequence of the small number of initial ESTs corresponding to immature seeds (11,032 sequences). This is especially true in the case of genes only expressed during very early stages of seed development, for which only 232 ESTs are available. A recent report showed that only 16,115 of Arabidopsis genes are represented in the EST databases [35]. An additional problem is that not all the genes are represented in the Affymetrix 22K GeneChip^®^. We estimate that, if all genes were present in EST and microarray databases about a hundred would have been selected by our *in silico *method. It has been proposed that the developmental processes occurring during embryogenesis are active during the vegetative development of the plant, therefore some genes may also be expressed in other growing organs of the plant, and so not seed specific.

### Functional classification of the selected genes

The 49 selected seed-specific genes were grouped into different functional categories (Table 2) according to their predicted gene products, based on the Gene Ontology (GO) Consortium through the Arabidopsis consortium information [28]. The data were compared with the functional categories assigned for all Arabidopsis genes [36].

14 of the selected genes correspond to genes of unknown function (28.6%). This is lower but not significantly different (Fisher's exact test, α = 0.05) to the percentage obtained for the total genome (38.4%). Particularly interesting is At1g62060, whose function is unknown but is represented in databases by a total of 57 EST sequences (32 from immature seed libraries). Two of the genes encode germin-like proteins (At3g04170 and At3g04190), and four have been listed as seed or embryo specific genes of unknown function (At1g67100, At3g12960, At5g07190 and At5g55240).

Genes in the "nutrient reservoir" category represent 20.4% of the selection and include ten genes, four encoding oleosins, three globulins, two cruciferins and one a patatin-like protein. Accumulation of seed storage proteins is a highly seed specific process [37], so it is not surprising that the proportion of these genes in the selected group is significantly higher than that obtained for the whole genome (0.2%).

The third category is "response to abiotic stress", which includes six genes (12.2%), and is significantly more abundant than in the whole genome (3.1%). This is an indication of the importance of genes providing stress-tolerance in correct seed development. Three of the genes encode oxidative stress-related enzymes, the function of two genes is related to desiccation (At3g62730 and At5g44310), and one is an ABA and stress inducible gene (At5g62490).

Five genes involved in carbohydrate metabolism were selected (10.2%). This percentage is significantly higher than that observed for the whole genome (2.4%). This category includes a gene encoding a xyloglucan:xyloglucosyl transferase (At3g48580), an enzyme (E.C.2.4.1.207) involved in the biosynthesis of the cell wall. It also includes a gene encoding a sucrose synthase (At5g49190). Sucrose represents a signal for differentiation during embryo development and up-regulates storage-associated gene expression [38].

Five genes involved in protein modification, localization or degradation were selected (10.2%), two of them being proteases (At3g54940 and At5g09640). No genes involved in translation were selected, even though these represent 2.7% of the genes in the whole genome, nor any involved in transport and subcellular trafficking, even though these represent 8.7% of the genes in the whole genome.

Four genes involved in different aspects of development (8%) were selected. Two of them are involved in cell wall synthesis or modification (At5g59170, encoding a cell wall protein precursor, extensin; and At3g60730, encoding a pectinesterase-like protein). This is an indication of the high rate of synthesis of new cell wall during seed development, and could also be an indication of the importance of specific cell wall components in co-ordinating gene expression programmes during embryo development [39], an effect observed in immature maize embryos [40]. The number of selected genes involved in development is not significantly higher than in the whole genome (60%). This is not surprising as the whole genome contains several genes involved, for example, in flower or root development. A third gene encodes an auxin-responsive GH3 family protein (At1g48660). Auxins are important signalling molecules involved in shoot/root axis establishment, among other processes [41].

Two genes involved in the regulation of gene expression (40%) were selected : *abi3 *and a gene encoding a CCCH-type zinc finger protein (At1g03790). Although not significantly, this number is lower than that observed for the whole genome (7.4%). The reduced number of transcription factor genes selected is surprising, but recent data from global analysis of gene expression indicate that the number of transcription factor genes specifically expressed during seed development is relatively low compared with other organs [8,42]. The expression of several MADS-box genes have been analyzed in different Arabidopsis tissues and it was found that, although many of these genes are expressed in embryonic tissue culture, few of them are exclusively expressed in this tissue [42]. Similarly, the number of specifically expressed transcription factor genes in developing siliques is relatively low compared to other tissues [8]. An additional explanation could be that, as this category of genes has relatively low levels of expression, they may be under-represented in EST collections used for selection.

Finally, two genes involved in respiration and energy (4.1%) and one in secondary metabolism (2.0%) (At1g14950 encoding a major latex protein type 1) were selected. Interestingly, two of the most highly represented categories in the genome are not represented in our selection: metabolism (6.4%) and transcription and splicing (6.1%). Nor were any genes detected for cell division, metabolism of amino acids, nucleic acid or lipids, defense or photosynthesis. As these genes are involved in general cell processes, they are expressed in several tissues and organs and they are unlikely to be selected in a seed-specific subtraction.

### Gene redundancy and mutant phenotypes

Mutational approaches have been extensively used in Arabidopsis to identify gene functions [3]. Mutation in about 800 genes produced loss of function phenotypes in Arabidopsis [6]. Of these, about 250 produce an altered embryo. Based on the information available in the Arabidopsis information resource (TAIR) [28] and Seedgenes [7], two of the 49 genes have a mutant phenotype (4%) (Table 1), and in only one of them the mutation produces alterations in embryo development (*abi3*). Gene redundancy may explain the reduced number of mutants detected. Many Arabidopsis genes are in tandem arrays or segmental duplications [43]. We examined how many of the genes in our selection were part of gene tandem arrays or duplicated in different parts of the genome (Table 1). 11 of the selected genes (22%) are duplicated, which is higher than that observed in the whole genome (17%) (p-value = 0.33 in Fisher's exact test).

### Patterns of gene expression during silique and seed development

**Figure 4:**
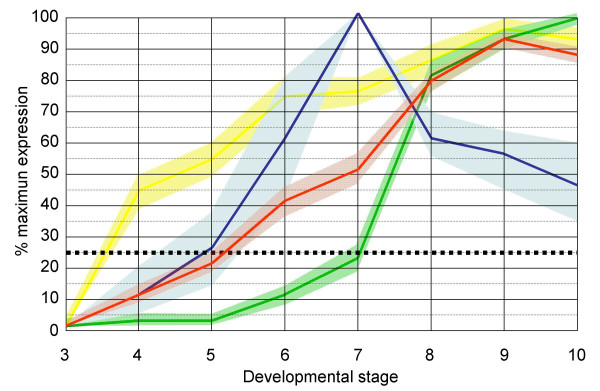
**Expression profiles during seed development showing four different patterns of expression in the subtracted genes. **Expression data are based on the microarray results [9]. Blue, pattern I; yellow, pattern IIa; red, pattern IIb; green, pattern IIc. Solid lines correspond to average expression and shaded areas to the standard errors. Developmental stages: 3, siliques with embryos at the mid-globular to early heart embryo stage; 4, siliques with embryos at the early to late heart-embryo stage; 5, siliques with embryos at the late heart to mid torpedo stages; 6, seeds with embryos at the late torpedo stage; 7, seeds with embryos at the late torpedo to early walking-stick stage; 8, seeds with embryos at the walking-stick to early curled-cotyledon stages; 9, seeds with embryos at the curled-cotyledon to early green-cotyledon stages; 10, seeds with embryos at the green cotyledon stage. The dotted line corresponds to 25% of the maximum expression.

The patterns of expression during seed development were investigated for each of the selected genes. Expression data was obtained from the Digital Northern tool in Genevestigator [24], corresponding to microarray hybridization of Affymetrix ATH1GeneChip^® ^microarrays using labelled cDNAs of siliques and seeds at different stages of development, from mid-globular to green cotyledon embryos [9]. We used SOTA analysis in the TMEV 3.1 analysis package to identify expression patterns during silique and seed development (Figure 4). From this analysis, we can distinguish four major patterns of expression (Table 1):

Group I: higher expression at early seed development. Genes that reach the maximum level of expression between late torpedo and early walking-stick embryo stages. This group includes five genes: At5g09640, encoding a serine carboxypeptidase, At5g49190, encoding a sucrose synthase, At2g34700, encoding a proline rich glycoprotein, and two genes encoding germin-like proteins (At3g04170 and At3g04190).

Group II: higher expression at mid seed development or later. The expression increases progressively, reaching the maximum level at the early cotyledon stage or later. In turn, SOTA analysis divided this class into three groups that can be distinguished by the stage at which their transcription level is higher than 25% of the maximum:

• IIa. Very early expression. The expression increases to more than 25% of the maximum before the early embryo stage. Four genes are included in this group. At5g48100, encoding a laccase, At4g36700, encoding a globulin-like protein, At4g37050, encoding a patatin-like protein, and At1g62060, encoding a protein of unknown function.

• IIb. Early expression. The expression increases to more than 25% of the maximum between the early heart and late torpedo stages. This group has 23 genes and includes the majority of the "nutrient reserve" genes.

• IIc. Mid stage expression. The expression increases to more than 25% of the maximum later than the late torpedo stage. It includes 17 genes of diverse functions.

## Conclusion

Despite the technical problems associated with the relatively reduced number of Arabidopsis ESTs available, we have demonstrated here that the combination of EST profiling with microarray-based *in silico *selection may be a quick and cheap first step in the identification of Arabidopsis genes specifically expressed in certain organs, or in response to certain environmental stimuli. The same method could be applied to several other plant species in which EST sequences are available from several different organs and under different conditions (maize, wheat, rice, barley soybean, loblolly pine, etc). However, microarray data available for species other than Arabidopsis are very limited and less openly accessible, severely limiting the applicability of our two-step selection approach. An increase in EST sequencing, using more specific libraries, and in the contents of public microarray databases will greatly contribute to the efficiency of the method in plants.

## Methods

### Plant material

*Arabidopsis thaliana *Col-0 plants were grown in soil, in growth chambers, at 22°C, with 18 h day. Plants used for root RNA extractions were grown on 0.8% (w/v) MS basal salt mixture agar plates in growth chambers, at 22°C, with 18 h day.

### cDNA library construction and tag sequencing of expressed sequences

Total RNA was extracted from frozen seeds as previously described [44] and treated with RNAse-free DNAseI (Promega). Double stranded cDNA was built using the SMART cDNA Library Construction Kit (Clontech) according to the manufacturer's instructions, and introduced into the pCRII-TOPO (Invitrogen) vector for sequencing using the TOPO TA Cloning kit (Invitrogen).

For sequencing, DNA was amplified using PCR primers specific for the plasmid vector (5'-GTCACGACGTTGTTAAACGACGGC-3' and 5'-GGAAACAGCTATGACCATGATTACG-3') and sequencing was carried out using a 5' specific primer (5'-GTATCAACGCAGAGTCG-3') and BigDye Terminator (Applied Biosystems) technology according to the manufacturer's instructions, in an ABI PRISM 3700 (Applied Biosystems). Cloning vector sequences were masked, and low quality and short (<190 bp) sequences removed. Homology searches for function assignment were performed using the BLASTN program in the Arabidopsis Information Resource (TAIR) [28]. EST sequences were deposited in the GeneBank database under the Accession numbers AM111128-AM111305.

### *In Silico *Subtraction

Newly sequenced expressed sequence tags and 10,854 EST sequences of three libraries from immature *Arabidopsis *seeds (5564, 5576 and #C6I in TIGR Arabidopsis Gene Index [29] were used as the initial source of immature seed sequences. *In silico *subtraction was done using a second set of EST libraries that did not contain immature seed sequences (50,992 ESTs from 78 libraries). Comparisons were based on the tentative gene contigs classification in the TIGR Arabidopsis database [29]. Libraries constructed from mixed tissues which could include immature seeds, such as immature siliques, were not considered for the subtraction. Subtraction was done by comparing the lists of genes that are represented in "immature seed" EST libraries with the list of genes represented by in "other organ" EST libraries.

A second selection step was based on the Arabidopsis Affymetrix GeneChip^® ^data, available from the Meta-analyzer tool of the Genevestigator software [24-26]. Genes represented in the arrays with more than one probe were selected only when the results with all the probes passed the selection criteria.

### Gene Ontology

Functional characterization was performed according to the Gene Ontology (GO) Consortium through the Arabidopsis consortium information [28]. Fisher's exact test was performed using the MATFORSK, Norwegian Food Research Institute online facility [45,46].

### RT-PCR

Total RNAs were extracted from frozen organs of Arabidopsis as previously described [44] and treated with RNAse-free DNAseI (Promega). Total pre-treated RNA (2 μg) was reverse transcribed with the Omniscript reverse transcriptase kit (Qiagen) using an oligo-dT primer. cDNAs were amplified with specific primers (Table 3), and controls, with non-reverse transcribed RNA, were also used to detect gDNA contamination. The actin gene was used as a control for RNA loading. PCR reactions were performed using 0.2 mM of each dNTP, 360 μg/ml BSA and 1 pmol μL-1 of each primer in a final volume of 50 μL. The reaction mixtures were heated to 95°C for 5 min, followed by 28 cycles of 94°C for 30 sec, 55°C for 30 sec, and 72°C for 90 sec. Reactions were completed by incubating at 72°C for 10 min. The amounts of template cDNA and the number of PCR cycles were determined for each gene to ensure that amplification occurred in the linear range and allowed for good comparison of the amplified products. At least two independent analyses were carried out on the different RNA samples. Reactions were performed in a Minicycler (MJ Research, Waltham, MA) thermal cycler.

### *In situ *hybridization

The protocol for *in situ *hybridization was done as previously described [47] except for the labelling of the probes and the detection of the signal. Probes were synthesized and labelled using the Boehringer digoxigenin system, and detected using the BM purple AP substrate (Boehringer). The probe was synthesized from the product of PCR amplification cloned into the pCRII-TOPO vector (Invitrogene).

### Gene distribution in tandem arrays and mutants

The presence of the selected genes in tandem arrays was based on previously described data [43]. Genes whose loss-of-function give an embryo mutant phenotype were determined according to data previously collected [6,7].

### Expression cluster analysis

For expression cluster analysis, we used the TIGR Multi Experiment Viewer (TMEV) software [48]. Original data was obtained from the Genevestigator tool [24-26] and correspond to a microarray analysis of silique and seed development [9].

## Authors' contributions

CB carried out the experimental molecular genetic studies. Database searches and analyses were performed by CMV and CB. PP supervised the study and wrote the manuscript jointly with CMV and CB.

## Supplementary Material

Additional file 1 **Libraries used in the subtraction process step 1 **Data obtained from the TIGR Arabidopsis Gene Index . Click here for file

Additional file 2 **Genes selected by EST subtraction **Genes having corresponding EST sequences in immature seed libraries and not in libraries of other tissues. Click here for file

## Figures and Tables

**Table 1:** Genes selected by *in silico *subtraction

Gene AGI code	Imm. seed ESTs	Indi- ferent ESTs	Definition	Functional category	Pattern of expression^1^	Mutants	Tandem arrays	Segmental duplication
At1g03790	1	5	Zinc finger (CCCH-type) family protein	Regulation of gene expression	IIc	-	1	1
At1g03890	41	22	Cruciferin 12S seed storage protein	Nutrient reservoir	IIb	-	2	1
At1g14950	2	15	Major latex protein type1	Secondary metabolism	IIc	-	4	1
At1g48130	2	12	Peroxiredoxin	Response to abiotic stress	IIb	-	1	1
At1g48660	1	0	Auxin-responsive GH3 family protein	Development	IIc	-	3	1
At1g62060	32	25	Unknown	Unknown	IIa	-	2	1
At1g65090	2	6	Unknown	Unknown	IIb	-	1	1
At1g67100	3	3	Seed specific protein Bn15D17A	Unknown	IIb	-	1	1
At1g73190	8	16	Tonoplast intrinsic protein 3.1	Protein processing	IIb	-	1	2
At1g80090	3	2	Unknown	Unknown	IIb	-	1	1
At2g28420	1	4	Lactoylglutathione lyase family protein	Carbohydrate metabolism	IIc	-	1	1
At2g33520	1	1	Unknown	Unknown	IIc	-	1	1
At2g34700	2	4	Proline-rich glycoprotein	Development	I	-	1	1
At3g01570	15	52	Oleosin	Nutrient reservoir	IIb	-	1	1
At3g04170	1	0	Germin-like protein subfamily 1	Unknown	I	-	5	1
At3g04190	1	0	Germin-like protein subfamily 1	Unknown	I	-	5	1
At3g12960	1	0	Similar to seed maturation protein PM28	Unknown	IIc	-	1	1
At3g24650	6	3	ABI3 protein	Regulation of gene expression	IIb	Abi3^2^	1	1
At3g27660	7	0	Oleosin	Nutrient reservoir	IIb	-	1	1
At3g48580	1	1	Xyloglucan:xyloglucosyl transferase	Carbohydrate metabolism	IIc	-	1	1
At3g54940	4	18	Cysteine proteinase	Protein processing	IIb	-	1	1
At3g60730	2	2	Pectinesterase-like protein	Development	IIc	-	1	1
At3g61040	1	0	Cytochrome P450 monooxygenase-like	Respiration and energy	IIc	-	1	1
At3g62730	55	17	Desiccation-related protein	Response to abiotic stress	IIb	-	1	1
At3g63040	1	0	Unknown	Unknown	IIb	-	1	1
At4g25140	1	5	Glycine-rich protein/ oleosin	Nutrient reservoir	IIb	-	1	1
At4g27150	68	33	2S seed storage protein 2 precursor	Nutrient reservoir	IIb	-	4	1
At4g28520	92	4	12S cruciferin seed storage protein (CRU3)	Nutrient reservoir	IIb	-	1	1
At4g36700	48	16	Globulin-like protein	Nutrient reservoir	IIa	-	1	1
At4g37050	2	5	Patatin-like	Nutrient reservoir	IIa	-	3	1
At5g01670	1	1	Aldose reductase-like protein	Carbohydrate metabolism	IIc	-	1	1
At5g03860	1	18	Malate synthase	Carbohydrate metabolism	IIc	-	1	1
At5g04010	1	0	Unknown	Unknown	IIc	-	1	1
At5g07190	10	15	Embryo-specific protein 3 (ATS3)	Unknown	IIb	-	1	1
At5g09640	10	4	Serine carboxypeptidase-like	Protein processing	I	Sng2^3^	1	1
At5g22470	8	1	Poly (ADP-ribose) polymerase family protein	Protein processing	IIc	-	1	1
At5g40420	39	68	Oleosin	Nutrient reservoir	IIb	-	1	1
At5g44310	5	1	Late embryogenesis abundant protein-like	Response to abiotic stress	IIc	-	1	1
At5g45690	4	6	Unknown	Unknown	IIc	-	1	1
At5g45830	1	1	Unknown	Unknown	IIc	-	1	1
At5g48100	30	19	Laccase	Response to abiotic stress	IIa	-	1	1
At5g49190	9	0	Sucrose synthase (SUS2)	Carbohydrate metabolism	I	-	1	1
At5g50700	9	41	11-beta-hydroxysteroid dehydrogenase-like	Response to abiotic stress	IIb	-	2	1
At5g54740	7	37	2S storage protein-like	Nutrient reservoir	IIb	-	1	1
At5g55240	6	3	Embryo-specific protein 1	Unknown	IIb	-	1	1
At5g57260	1	0	Cytochrome P450	Respiration and energy	IIb	-	1	2
At5g59170	11	6	Cell wall protein precursor, extensin	Development	IIb	-	1	1
At5g62490	2	5	AtHVA22b	Response to abiotic stress	IIc	-	1	1
At5g62800	1	0	Seven in absentia (SINA) family protein	Protein processing	IIb	-	1	1

(1) Information in Figure 4.

(2) Mutant is abscisic acid-insensitive and lacks seed dormancy.

(3) Mutant accumulates sinapoylglucose instead of sinapoylcholine.

**Table 2:** Functional categories of the seed specific genes

**Functional category**	**Whole genome (%)**	**Subtracted genes (%) (p-value)**^1^
Amino acid metabolism	0.1	0.0^1.00^
Carbohydrate metabolism	2.4	10.2^0.01*^
Cell division cycle	2.3	0.0^0.63^
Defense	0.9	0.0^1.00^
Development	6.0	8.2^0.54^
Lipid metabolism	0.9	0.0^1.00^
Metabolism	6.4	0.0^0.07^
Nucleic acid metabolism	3.1	0.0^0.41^
Nutrient reservoir	0.2	20.4^0.00*^
Photosynthesis	0.3	0.0^1.00^
Protein processing	9.4	10.2^0.81^
Regulation of gene expression	7.4	4.1^0.58^
Respiration and energy	4.0	4.1^1.00^
Response to abiotic stress	3.1	12.2 ^0.00*^
Secondary metabolism	0.7	2.0^0.28^
Transport and subcellular trafficking	8.7	0.0^0.02*^
Transcription and splicing	6.1	0.0^0.07^
Translation	2.7	0.0^0.64^
Unknown	38.4	28.6^0.17^

1. p-value for the same or a stronger association of Fisher's exact test compared with total genome

^*^. p-value < 0.05.

**Table 3:** Primers used for RT-PCR analysis

Gene (Atg)	Forward primer	Reverse primer
At5g09640	GACACACCAAACATCAGAACCG	CTACTCATCATCCAAGGTCTCC
At5g22470	TATGCTCTCTTCCGGTTCCTGG	ATGGAACCAACCGTCCACAAGG
At5g45690	ACGATTGCGACTCCTCTAAACC	GAACGGAGCCAATTTCTGCATC
At1g67100	GCTCATGAACCTCCTCAACACC	CCCGATCCAAGTCTTTGGTTCC
At3g60730	TCAAGCTGTGGCGTTGAGAGTG	GGTAAACGGAGAAGCCTCTTCC
At3g12203	GGCACTGATCTCTGATGAACAC	TTCTGAACCATCCATGGTCTCC
At1g71691	GCTTGTTCTTCATCGGAATGGG	TACGACAAGGCGTTTCAAAGGG
At2g43260	TTCCGGCTTGAACCATAACTGC	TGAACCACCTTTTCTGCCTTCG
At1g68380	TGTTTTATGGCCGCCGTATTCC	TCCAAGTAAGCGTCCTATTCGC
At4g14780	TCAAACTCGCTCTTGATCTCGC	TTTCACCACCTCCTTCATCTCC
