# Supplementary material for: Computational and experimental analysis identifies Arabidopsis genes specifically expressed during early seed development
Source: BMC Genomics. 2006 Feb 28;7:38. doi: 10.1186/1471-2164-7-38 (PMC1420293; doi:10.1186/1471-2164-7-38)
Supplement: Additional file 1 — Libraries used in the subtraction process step 1 Data obtained from the TIGR Arabidopsis Gene Index . [file 1471-2164-7-38-S1.doc]

**Supplementary data 1 – Libraries used in the subtraction process step 1**

Data obtained from the TIGR Arabidopsis Gene Index (<http://www.tigr.org/tigr-scripts/tgi/T_index.cgi?species=arab>).

1. Immature seed

| **Cat#** | **Library Name** | **Supplier** | **Organ** | **Stage** | **Total ESTs** |
| --- | --- | --- | --- | --- | --- |
| 5564 | Arabidopsis developing seed | Michigan State University | seed | 5-13 days after flowering | 10800 |
| 5576 | A. thaliana early developing seeds | Columbia | Immature seeds | Heart stage | 9 |
| #C6I | Early Ovule Development Forward Subtracted | Penn State University | Ovule |  | 45 |
| - | Immature seeds | CSIC-IRTA | Immature seed | 2-6 days after pollination | 178 |

1. Other tissues

| **Cat#** | **Library Name** | **Supplier** | **Organ** | **Stage** | **Total ESTs** |
| --- | --- | --- | --- | --- | --- |
| **Dry seeds** | | | | | |
| NH14 | Perp-dry-seedA | Universite de Perpignan | Dry seed | Dry seed | 346 |
| **Leaf and stem** | | | | | |
| #EP3 | Arabidopsis thaliana aerial vegetative tissues 4-weeks old | URGV,  CNRS/ INRA | aerial vegetative tissues | 4-weeks old | 84 |
| #F0G | Arabidopsis thaliana Adult vegetative tissue Col-0 | Genoscope | Adult vegetative tissue | Adult | 2416 |
| #DGL | Arabidopsis Leaf Senescence Library | Cornell University | Leaf | Rosette leaf | 5419 |
| 2338 | A. thaliana, Columbia Col-0, rosette-1 | Columbia | Rosette | 4-7 weeks | 611 |
| 2340 | A. thaliana, Columbia Col-0, rosette-2 | Columbia | Rosette | 4-7 weeks | 874 |
| 2341 | A. thaliana, Columbia Col-0, rosette-3 | Columbia | Rosette | 4-7 weeks | 785 |
| NH29 | shoots, 2 weeks old | Institute de Biotechnologie des Plantes, Paris | Shoot | 2 week old | 1 |
| 5335 | Arabidopsis thaliana aboveground organs two to six-week old | Kazusa DNA Research Institute | Aboveground organ | 2-6 week old | 12264 |
| 3792 | Arabidopsis thaliana Above-ground organ from two to six-week old plants Columbia | Kazusa DNA Research Institute | Aboveground organ | 2-6 week old | 17 |
| 4063 | Arabidopsis thaliana above-ground organ two to six-week old | Kazusa DNA Research Institute | Aboveground organ | 2-6 week old | 3348 |
| #A5R | RAFL12 | RIKEN | Leaves | Rosette | 17 |
| #A5S | RAFL13 | RIKEN | Leaves | Rosette | 64 |
| 4921 | AB | Columbia | Leaf, seedling | 2-3 weeks | 642 |
| NH25 | seedling hypocotyl | Michigan State University | Seedling | 3 day-old | 453 |
| NH26 | seedling hypocotyl | Michigan State University | Seedling | 3 day-old | 819 |
| NH27 | seedling hypocotyl | Michigan State University | Seedling | 3 day-old | 563 |
| NH28 | seedling hypocotyl | Michigan State University | Seedling | 3 day-old | 724 |
| 4932 | AA | Columbia | Leaf, flowering plants | 12 weeks | 1007 |
| #BU2 | Arabidopsis thaliana Differential Display | ARS/USDA | Leaf | Rosette | 5 |
| NH12 | Ors-A | Institute de Biotechnologie des Plantes, Paris | Green shoots | Green shoots | 322 |
| NH16 | Strasbourg-A | CNRS | Leaf | leaf strips incubated 2/3/4 days in liquid culture medium | 667 |
| NH17 | Strasbourg-FA | CNRS | Leaf | leaf strips incubated 2/3/4 days in liquid culture medium | 37 |
| **Root** | | | | | |
| #DOU | Hairy root culture | Commonwealth Scientific and Industrial Research Organisation | Root | Cultured adult roots | 4 |
| 2336 | A. thaliana Col-0, root-1 | Columbia | Root | 4 - 7 weeks | 538 |
| 2337 | A. thaliana Col-0, root-2 | Columbia | Root | 4 - 7 weeks | 645 |
| 5336 | A. thaliana roots Columbia | Kazusa DNA Research Institute | Root |  | 17574 |
| #A5T | RAFL14 | RIKEN | Root |  | 24481 |
| #BMJ | Size-selected small cDNAs of A. thaliana | Delaware Biotechnology Institute | Root | 2 week | 20 |
| #E3R | MPIZ-ADIS-066 | Max Planck Institute | Root | 3 week | 1434 |
| NH13 | Ors-B | Institute de Biotechnologie des Plantes, Paris |  | Cultured adult roots | 239 |
| **Flower** | | | | | |
| #F0J | A. thaliana Flowers and buds Col-0 | Genoscope | Flower |  | 1724 |
| 2334 | A. thaliana, Col-0, inflorescence-1 |  |  |  | 356 |
| 2335 | A. thaliana Col-0, inflorescence-2 |  |  |  | 674 |
| 5337 | A. thaliana flower buds Columbia | Kazusa DNA Research Institute | Flower | Flower buds | 5719 |
| NH08 | Grenoble-A; flower buds | CNRS | Flower | Flower buds | 2 |
| NH09 | Grenoble-B; flower buds | CNRS | Flower | Flower buds | 984 |
| #E55 | A. thaliana ag-1 35S:AG-GR forward subtraction library | The Pennsylvania State University | Inflorescence lacking open or older flowers | 4 week | 1447 |
| #EA9 | AtM1 | Max Planck lnstitute | Inflorescence meristem | 1 week after bolting | 4382 |
| NH36 | CD4-6 | Michigan State University | Inflorescence with predominately young flower buds |  | 201 |
| NH35 | pi mRNA differential display clones | University of Auckland | Inflorescence | < stage 9 | 20 |
| #CCH | MPIZ-ADIS-035 | Max Planck lnstitute | Inflorescence |  | 1165 |
| **Biotic stres** | | | | | |
| #BKG | Infected Arabidopsis Leaf | Aalborg Universitet | E. cichoracearum infected leaf | Plant 3 weeks old, three days post infection | 3301 |
| #CAH | Arabidopsis avirulent Pseudomonas syringae subtracted library | Penn State University | Rosette leaf | 4 week old | 232 |
| #CAI | Arabidopsis virulent Pseudomonas syringae subtracted library | Penn State University | Rosette leaf | 4 week old | 140 |
| #C6J | Virulent Peronospora parasitica Infected Arabidopsis Forward-Subtracted Library | Penn State University | Leaf |  | 243 |
| #C6K | Avirulent Peronospora parasitica Infected Forward Subtracted Library | Penn State University | Leaf | 3-week old plants | 110 |
| #C6L | Virulent Peronospora parasitica Infected Arabidopsis reverse-Subtracted Library | Penn State University | Leaf |  | 11 |
| #C6M | Avirulent Peronospora parasitica Infected Reverse Subtracted Library | Penn State University | Leaf | 3-week old plants | 2 |
| 1725 | Cloned cDNAs of Arabidopsis thaliana generated by mRNA | Iowa State University | Excised pieces of roots containing nematode feeding | In vitro culture of adult vegetative plants | 22 |
| NH15 | Ra147.1 | CNRS/INRA | Pathogen-challenged cells |  | 121 |
| **Abiotic stress** | | | | | |
| #5GJ | Arabidopsis Acute Ozone Forward-Subtracted Library | Penn State University | Leaf |  | 170 |
| #5GK | Arabidopsis Acute Ozone Reverse-Subtracted Library | Penn State University | Leaf |  | 27 |
| #5GL | Arabidopsis Chronic Ozone Forward-Subtracted Library | Penn State University | Leaf |  | 128 |
| 6523 | AD A. thaliana (Col-0 gl1) library enriched for salt-induced transcripts | University of Nevada | Mixed leaf and root | 10-14 days old | 148 |
| 6524 | AD A. thaliana (Col-0 gl1) subtracted library enriched for salt-induced transcripts | University of Nevada | Mixed leaf and root | 10-14 days old | 536 |
| #C6P | Arabidopsis Acute Ozone pooled time-points Forward-Subtracted Library | Penn State University | Leaf |  | 142 |
| **Other** | | | | | |
| #CFQ | Cloned RT-PCR products of Arabidopsis thaliana | University of Arizona | Seedling | 7-10 days old | 80 |
| 2370 | Arabidopsis thaliana germinating seeds | Columbia | Germinating seeds |  | 16 |
| 4924 | AC | Columbia | Seedling, leaf and root | 2-3 weeks | 333 |
| 5338 | Arabidopsis thaliana liquid-cultured seedlings Columbia | Kazusa DNA Research Institute | Liquid-cultured seedlings |  | 1135 |
| NH18 | Versailles-VB | INRA | In vitro-grown etiolated seedlings | 5 days old | 1503 |
| NH19 | Versailles-VC | INRA | In vitro-grown etiolated seedlings | 5 days old | 124 |
| NH20 | Versailles-VD | INRA | In vitro-grown etiolated seedlings | 5 days old | 7 |
| #A64 | RAFL2 | RIKEN | Rosette plants |  | 433 |
| #A65 | RAFL3 | RIKEN | Rosette plants |  | 384 |
| #A66 | RAFL4 | RIKEN | Rosette plants |  | 1885 |
| #A67 | RAFL5 | RIKEN | Rosette plants |  | 3767 |
| #A69 | RAFL7 | RIKEN | Rosette plants |  | 3494 |
| #A6A | RAFL8 | RIKEN | Rosette plants |  | 3373 |
| #C6N | Methyl Jasmonate Treated Arabidopsis Reverse-Subtracted Library | Penn State University | Leaf |  | 3 |
| #C6H | Methyl Jasmonate Treated Arabidopsis Forward-Subtracted Library | Penn State University | Leaf |  | 56 |
| NH01 | AC13D | CNRS/INRA | Log cell suspension culture | Late log phase | 123 |
| NH02 | AC16H | CNRS/INRA | Cycling cell suspension culture | Cycling cells | 897 |
| #CAG | Arabidopsis Salicylic Acid Subtracted Library | Penn State University | Rosette leaf | 4 week-old | 107 |
| NH30 | Arabidopsis thaliana cell suspension | Univ. Paul Sabatier | Cell suspension |  | 27 |
| #F0H | Arabidopsis thaliana Hormone Treated Callus Col-0 | Genoscope | Callus | Hormone Treated Callus | 2308 |
| #CCG | MPIZ-ADIS-008 | Max Planck lnstitute | Seedling | Few days | 2390 |
