# Supplementary material for: Computational and experimental analysis identifies Arabidopsis genes specifically expressed during early seed development
Source: BMC Genomics. 2006 Feb 28;7:38. doi: 10.1186/1471-2164-7-38 (PMC1420293; doi:10.1186/1471-2164-7-38)
Supplement: Additional file 2 — Genes selected by EST subtraction Genes having corresponding EST sequences in immature seed libraries and not in libraries of other tissues. [file 1471-2164-7-38-S2.doc]

**Supplementary data 2 – Genes selected by EST subtraction**

Genes having corresponding EST sequences in immature seed libraries and not in libraries of other tissues.

| **AGI code or**  **GeneBank Acc.** | **TIGR code** | **Definicion** |
| --- | --- | --- |
| At1g01225 | TC267364 | NC domain-containing protein-related |
| At1g02740 | TC278236 | MRG family protein |
| At1g02770 | TC275927 | Unknown |
| At1g03103 | TC268770 | Protease inhibitor/seed storage/lipid transfer protein (LTP) family protein |
| At1g03106 | TC273344 | Unknown |
| At1g03210 | TC276036 | Similar to phenazine biosynthesis phzc/phzf family protein |
| At1g03560 | TC255717 | Pentatricopeptide (PPR) repeat-containing protein |
| At1g03790 | TC274104 | Zinc finger (CCCH-type) family protein |
| At1g03890 | TC251613 | Cruciferin 12S seed storage protein |
| At1g03920 | TC275977 | Protein kinase MK6 |
| At1g04160 | TC277571 | Myosin heavy chain MYA2 |
| At1g04880 | TC257963 | Glutathione S-transferase GST16-like |
| At1g05280 | TC278832 | Fringe-related protein |
| At1g05450 | TC255931 | Lipid-transfer protein putative |
| At1g06410 | TC263622 | Glycosyl transferase family 20 protein |
| At1g06450 | TC274674 | CCR4-NOT transcription complex protein |
| At1g06820 | TC256058 | Carotenoid isomerase |
| At1g07705 | TC266925 | VIP2 protein |
| At1g07950 | TC254374 | Surfeit locus protein 5 family protein |
| At1g08060 | TC263990 | MOM1 |
| At1g08420 | TC267099 | Serine/threonine phosphoesterase family protein |
| At1g08810 | TC274237 | MYB family transcription factor |
| At1g09155 | TC276278 | SKP1 interacting partner 3-related |
| At1g09190 | TC257368 | Pentatricopeptide (PPR) repeat-containing protein |
| At1g09380 | TC263149 | Putative nodulin protein N21 |
| At1g09490 | TC263673 | Alcohol dehydrogenase |
| At1g09550 | TC268517 | Pectinacetylesterase |
| At1g09580 | TC267661 | Transmembrane protein Tmp21 precursor |
| At1g09790 | TC267949 | COBRA-like protein 6 precursor |
| At1g09950 | TC274829 | Transcription factor-related |
| At1g10120 | TC268790 | Basic helix-loop-helix (bhlh) family protein |
| At1g10210 | TC254539 | Mitogen-activated protein kinase homolog 1 (MAP kinase 1) (atmpk1) |
| At1g10520 | TC267641 | DNA polymerase lambda |
| At1g10640 | TC269021 | Polygalacturonase |
| At1g10750 | TC275636 | Putative carboxyl-terminal peptidase |
| At1g11170 | TC253269 | Unknown |
| At1g11190 | TC262545 | Bifunctional nuclease (BFN1) |
| At1g11590 | TC255226 | Putative pectin methylesterase |
| At1g11720 | TC257480 | Starch synthase |
| At1g11960 | TC257361 | Early-responsive to dehydration protein-related |
| At1g12230 | TC278590 | Transaldolase-like protein |
| At1g12550 | TC265059 | Oxidoreductase family protein |
| At1g12805 | TC257360 | Unknown |
| At1g14260 | TC265415 | Zinc finger (C3HC4-type RING finger) family protein |
| At1g14580 | TC259683 | Putative zinc finger protein |
| At1g14950 | TC252695 | Major latex protein type1 |
| At1g14970 | TC257070 | Unknown |
| At1g15150 | TC254873 | MATE efflux family protein |
| At1g15200 | TC254630 | Protein-protein interaction regulator family protein |
| At1g15330 | TC273248 | CBS domain-containing protein |
| At1g15510 | TC268319 | Pentatricopeptide (PPR) repeat-containing protein |
| At1g16040 | TC277578 | Phosphatidylinositol-glycan biosynthesis class F protein |
| At1g16980 | TC268239 | Alpha-trehalose-phosphate synthase |
| At1g17060 | TC256663 | Cytochrome P450-like protein |
| At1g17380 | TC268739 | Unknown |
| At1g17650 | TC255116 | 6-phosphogluconate dehydrogenase NAD-binding domain-containing protein |
| At1g18950 | TC258930 | Aminoacyl-trna synthetase |
| At1g20260 | TC270864 | Vacuolar ATP synthase subunit B |
| At1g20410 | TC264876 | Unknown |
| At1g20500 | TC275366 | 4-coumarate-coa ligase-like protein |
| At1g21710 | TC255556 | 8-oxoguanine DNA glycosylase |
| At1g21730 | TC256127 | Kinesin-related protein |
| At1g21740 | TC278038 | Unknown |
| At1g22020 | TC264213 | Glycine hydroxymethyltransferase |
| At1g23980 | TC256248 | RING zinc finger protein-like |
| At1g24430 | TC256521 | Similar to deacetylvindoline 4-O-acetyltransferase |
| At1g25054 | TC268745 | UDP-3-O-acyl N-acetylglycosamine deacetylase |
| At1g25470 | TC255477 | Subfamily B-6 of ERF/AP2 transcription factor family. |
| At1g26090 | TC257571 | Unknown |
| At1g26370 | TC275409 | RNA helicase |
| At1g26570 | TC264346 | UDP-glucose dehydrogenase |
| At1g26680 | TC257022 | Transcriptional factor B3 family protein |
| At1g27040 | TC263230 | Putative nitrate transporter |
| At1g27590 | TC276948 | Unknown |
| At1g27760 | TC271419 | Unknown |
| At1g27960 | TC279820 | Unknown |
| At1g28500 | TC258852 | Unknown |
| At1g28540 | TC275708 | Unknown |
| At1g29400 | TC271188 | Probable RNA-binding protein |
| At1g29750 | TC264672 | Similar to leucine-rich repeat transmembrane protein kinase |
| At1g30550 | TC259577 | Unknown |
| At1g31470 | TC257109 | Nodulin-related |
| At1g32260 | TC275047 | Unknown |
| At1g33050 | TC271867 | Unknown |
| At1g33420 | TC265403 | Phd finger family protein |
| At1g34210 | TC256464 | Somatic embryogenesis receptor-like kinase 2 |
| At1g34360 | TC256692 | Translation initiation factor 3 (if-3) family protein |
| At1g35140 | TC264710 | Probable phosphate-induced (phi-1) protein |
| At1g35510 | TC278500 | Unknown |
| At1g36160 | TC272263 | Similar to acetyl-coa carboxylase 2 |
| At1g43675 | TC277623 | Surfeit locus protein 5 family protein |
| At1g44110 | TC256992 | Mitotic cyclin a2-type |
| At1g45180 | TC276096 | ZINC FINGER protein |
| At1g48130 | TC262619 | Peroxiredoxin |
| At1g48660 | TC258441 | Auxin-responsive GH3 family protein similar to auxin-responsive GH3 product |
| At1g50950 | TC268815 | Thioredoxin-related |
| At1g54080 | TC261325 | Oligouridylate binding protein putative partial |
| At1g54150 | TC274921 | Zinc finger |
| At1g54510 | TC265231 | Protein kinase family protein |
| At1g55080 | TC274231 | Unknown |
| At1g55270 | TC272209 | Kelch repeat-containing f-box family protein |
| At1g55940 | TC277513 | Cytochrome p450 |
| At1g56180 | TC264385 | Unknown |
| At1g60987 | - | Cysteine rich protein |
| At1g61690 | TC274482 | Tetratricopeptide repeat (TPR)-containing protein |
| At1g61720 | TC264032 | Dihydroflavonol 4-reductase |
| At1g61810 | TC275992 | Glycosyl hydrolase family 1 protein |
| At1g61820 | TC263171 | Glycosyl hydrolase family 1 protein |
| At1g61860 | TC257044 | Protein kinase |
| At1g62000 | TC261464 | Unknown |
| At1g62060 | TC261285 | Unknown |
| At1g62080 | TC261521 | Unknown |
| At1g62220 | TC254029 | Unknown |
| At1g62225 | TC271828 | Unknown |
| At1g62860 | TC278466 | Protein kinase |
| At1g62880 | TC273537 | Cornichon family protein |
| At1g63020 | TC266722 | RNA polymerase IIA largest subunit putative |
| At1g63140 | TC255296 | Caffeic O-methyltransferase |
| At1g63140 | TC255297 | O-methyltransferase |
| At1g63370 | TC274790 | Flavin-containing monooxygenase family protein |
| At1g63650 | TC264217 | Transcription factor EGL1 (Basic helix-loop-helix protein 2) |
| At1g64490 | TC266621 | Unknown |
| At1g64580 | TC274426 | Pentatricopeptide (ppr) repeat-containing protein |
| At1g65090 | TC264499 | Unknown |
| At1g65880 | TC265191 | Amp-dependent synthetase and ligase family protein |
| At1g66460 | TC275597 | Protein kinase-like protein |
| At1g67100 | TC272916 | Similar to seed specific protein Bn15D17A |
| At1g67420 | TC268852 | Peptidase family-like protein |
| At1g68120 | TC267291 | Unknown |
| At1g68170 | TC278190 | Mtn21-like protein |
| At1g68380 | TC274078 | Unknown |
| At1g68460 | TC266602 | Cytokinin synthase |
| At1g69040 | TC253973 | Act domain containing protein |
| At1g69570 | TC264709 | Dof-type zinc finger domain-containing protein |
| At1g69670 | TC256753 | Cullin 3B |
| At1g70895 | TC267074 | Clavata3/esr-related 17 |
| At1g71120 | TC257705 | Gdsl-motif lipase/hydrolase family protein |
| At1g71250 | TC255820 | GDSL-motif lipase/hydrolase/hydrolase |
| At1g71691 | TC273517 | GDSL-motif lipase/hydrolase/hydrolase |
| At1g71950 | TC278289 | Unknown |
| At1g72190 | TC256049 | Phosphoglycerate dehydrogenase |
| At1g72270 | TC276544 | Unknown |
| At1g72560 | TC257501 | Trna export mediator exportin-t |
| At1g72600 | TC276178 | Hydroxyproline-rich glycoprotein family protein |
| At1g72670 | TC276961 | Calmodulin-binding family protein |
| At1g72830 | TC253699 | CCAAT-binding factor B subunit-like protein |
| At1g73190 | TC252299 | Tonoplast intrinsic protein 3.1 |
| At1g73290 | TC257943 | Serine carboxypeptidase |
| At1g73550 | TC267238 | Lipid transfer protein |
| At1g75020 | TC258136 | Phospholipid/glycerol acyltransferase family protein |
| At1g75860 | TC276433 | Unknown |
| At1g76110 | TC254387 | High mobility group (hmg1/2) |
| At1g76290 | TC277723 | Amp-dependent synthetase and ligase family protein |
| At1g76630 | TC267677 | Tetratricopeptide repeat (tpr)-containing protein |
| At1g76880 | TC274329 | DNA-binding protein DF1 |
| At1g77300 | TC269040 | SET domain-containing protein |
| At1g78390 | TC266589 | Putative 9-cis-epoxycarotenoid dioxygenase |
| At1g78540 | TC259602 | Transcription factor-related |
| At1g79430 | TC254186 | Myb DNA binding protein |
| At1g79690 | TC276846 | Mutt/nudix family protein |
| At1g79840 | TC264438 | Homeobox protein GLABRA2 |
| At1g79950 | TC276691 | Helicase-related |
| At1g80090 | TC278341 | CBS domain-containing protein |
| At1g80370 | TC258460 | Cyclin |
| At2g01130 | TC265554 | Helicase domain-containing protein |
| At2g01610 | TC266479 | Invertase/pectin methylesterase inhibitor family protein |
| At2g01750 | TC275097 | Microtubule associated protein |
| At2g02120 | TC264415 | Plant defensin-fusion protein |
| At2g02490 | - | Proline-rich family protein |
| At2g03390 | TC258931 | Uvrb/uvrc motif-containing protein |
| At2g03780 | TC256169 | Translin family protein |
| At2g04039 | TC275315 | Unknown |
| At2g05760 | TC276479 | Xanthine/uracil permease family protein |
| At2g07170 | TC277832 | Unknown |
| At2g07718 | TC257239 | Cytochrome b |
| At2g14680 | TC267562 | Myosin heavy chain-related |
| At2g17300 | TC267464 | Unknown |
| At2g18160 | TC261643 | Bzip transcription factor family protein |
| At2g18850 | TC275254 | Set domain-containing protein |
| At2g18915 | TC265884 | E3 ubiquitin ligase scf complex f-box subunit |
| At2g20310 | TC267539 | Unknown |
| At2g20440 | TC266613 | Rabgap/tbc domain-containing protein |
| At2g20770 | TC274172 | Lanthionine synthetase c-like family protein |
| At2g21185 | TC263681 | Unknown |
| At2g21260 | TC273647 | NADPH dependent mannose 6-phosphate reductase |
| At2g22030 | TC267317 | Kelch repeat-containing f-box family protein |
| At2g22560 | TC277813 | Kinase interacting protein-related |
| At2g22570 | TC278498 | Isochorismatase hydrolase family protein |
| At2g22910 | TC256168 | Putative amino acid acetyltransferase |
| At2g23140 | TC256913 | Armadillo/beta-catenin repeat family protein |
| At2g23550 | TC266043 | Acetone-cyanohydrin lyase |
| At2g23560 | TC278169 | Acetone-cyanohydrin lyase |
| At2g23580 | TC273923 | Acetone-cyanohydrin lyase |
| At2g24640 | TC255345 | Ubiquitin carboxyl terminal hydrolase |
| At2g25940 | TC254259 | Vacuolar processing enzyme alpha-isozyme precursor (Alpha-VPE) |
| At2g26580 | TC274658 | Plant-specific transcription factor yabby family protein |
| At2g27240 | TC268181 | Unknown |
| At2g27775 | TC279724 | Unknown |
| At2g28240 | TC265858 | Putative hydroxyproline-rich glycoprotein |
| At2g28420 | TC274367 | Lactoylglutathione lyase family protein / glyoxalase I family protein |
| At2g28650 | TC274204 | Exocyst subunit exo70 family protein |
| At2g30300 | TC277331 | Nodulin-related |
| At2g30350 | TC267880 | Endo/excinuclease amino terminal domain |
| At2g30615 | TC256068 | Unknown |
| At2g30680 | TC268036 | Similar to glycosyl transferase family 48 protein |
| At2g30800 | TC264682 | ATP-dependent RNA helicase A |
| At2g30942 | TC269210 | Unknown |
| At2g32330 | TC259172 | Unknown |
| At2g32670 | TC265205 | Synaptobrevin family protein |
| At2g33240 | TC268808 | Myosin |
| At2g33520 | TC275699 | Unknown |
| At2g33580 | TC259106 | Peptidoglycan-binding lysm domain-containing protein kinase |
| At2g33690 | TC276402 | Late embryogenesis abundant protein |
| At2g34700 | TC264743 | Extensin family protein |
| At2g35530 | TC268009 | Bzip transcription factor family protein |
| At2g36400 | TC274405 | Transcription activator grl3 |
| At2g36760 | TC277238 | Udp-glucoronosyl/udp-glucosyl transferase family protein |
| At2g37025 | TC256715 | Pathogen-responsive dna-binding protein-related |
| At2g37090 | TC272851 | Glycosyltransferase |
| At2g38090 | TC254271 | MYB transcription factor |
| At2g38590 | TC267548 | F-box family protein |
| At2g38740 | TC273682 | Haloacid dehalogenase-like hydrolase family protein |
| At2g38920 | TC275414 | Spx (syg1/pho81/xpr1) domain-containing protein |
| At2g39560 | TC256033 | Unknown |
| At2g39680 | TC265171 | Trans-acting sirna primary transcript |
| At2g40150 | TC265866 | Unknown |
| At2g41070 | TC252623 | Bzip protein DPBF4 |
| At2g41140 | TC276380 | Calcium-dependent protein kinase |
| At2g41540 | TC262537 | Glycerol-3-phosphate dehydrogenase |
| At2g41700 | TC254835 | ATP-binding cassette transporter atabca1 |
| At2g41880 | TC275970 | Guanylate kinase 1 |
| At2g42110 | TC276810 | Unknown |
| At2g42170 | TC276214 | Actin 2 |
| At2g42950 | TC265979 | Unknown |
| At2g43190 | TC255068 | Ribonuclease p family protein |
| At2g43260 | TC254993 | F-box family protein |
| At2g43400 | TC262174 | Electron transfer flavoprotein-ubiquinone oxidoreductase family protein |
| At2g43880 | TC276244 | Polygalacturonase |
| At2g44470 | TC254864 | Putative beta-glucosidase |
| At2g45250 | TC276816 | Unknown |
| At2g45420 | TC265771 | LOB domain protein 18 |
| At2g45900 | TC277822 | Unknown |
| At2g46550 | TC262497 | Unknown |
| At2g46960 | TC252957 | Cytochrome P450-like protein |
| At2g47120 | TC278076 | Short-chain dehydrogenase/reductase (sdr) family protein |
| At2g47750 | TC273105 | Auxin-regulated protein GH3 homolog |
| At3g01160 | TC265914 | Unknown |
| At3g01570 | TC251579 | Oleosin |
| At3g02890 | TC257489 | Phd finger protein-related |
| At3g03020 | TC277593 | Unknown |
| At3g03240 | TC258420 | Esterase/lipase/thioesterase family protein |
| At3g03300 | TC266924 | Dead/deah box helicase carpel factory-related |
| At3g04170 | TC276999 | Germin-like protein subfamily 1 member 3 precursor |
| At3g04180 | TC255981 | Germin-like protein subfamily 1 member 4 precursor |
| At3g04190 | TC267823 | Germin-like protein subfamily 1 member 5 precursor |
| At3g04660 | TC258810 | F-box family protein |
| At3g04690 | TC268418 | Protein kinase family protein |
| At3g04960 | TC257772 | Unknown |
| At3g05400 | TC253408 | Putative sugar transporter |
| At3g05800 | TC257173 | Unknown |
| At3g07200 | TC273968 | ZINC FINGER family protein |
| At3g07730 | TC257774 | Unknown |
| At3g08900 | TC265817 | UDP-glucose:protein transglucosylase-like protein |
| At3g08910 | TC275769 | DNAJ heat shock protein |
| At3g08970 | TC255587 | Putative dnaj protein |
| At3g10400 | TC275282 | Rna recognition motif (rrm)-containing protein |
| At3g11020 | TC274803 | Dreb subfamily a-2 of erf/ap2 transcription factor family |
| At3g11180 | TC264077 | Leucoanthocyanidin dioxygenase-like protein |
| At3g11590 | TC274447 | Unknown |
| At3g11650 | TC257781 | Harpin-induced family protein |
| At3g12203 | TC264046 | Serine carboxypeptidase |
| At3g12550 | TC277084 | Xh/xs domain-containing protein |
| At3g12960 | TC267267 | Expressed protein similar to seed maturation protein PM28 |
| At3g13030 | TC264649 | Hat dimerisation domain-containing protein |
| At3g13640 | TC274297 | Rnase l inhibitor protein |
| At3g14130 | TC255339 | Glycolate oxidase |
| At3g14630 | TC266176 | Cytochrome p450 |
| At3g15050 | TC258786 | Calmodulin-binding family protein |
| At3g15510 | TC263196 | Atnac2 |
| At3g15680 | TC275230 | Zinc finger (ran-binding) family protein |
| At3g17030 | TC278454 | Unknown |
| At3g17680 | TC255787 | Unknown |
| At3g18570 | TC255057 | Oleosin |
| At3g18950 | TC265769 | Transducin family protein |
| At3g18970 | TC267906 | Pentatricopeptide (ppr) repeat-containing protein |
| At3g19260 | TC272922 | Longevity-assurance (lag1) family protein |
| At3g19500 | TC256280 | Ethylene-responsive protein -related |
| At3g19870 | TC275442 | Unknown |
| At3g20420 | TC256243 | Ribonuclease iii family protein |
| At3g20520 | TC273044 | Probable glycerophosphoryl diester phosphodiesterase 2 precursor |
| At3g20680 | TC274468 | Unknown |
| At3g20810 | TC274577 | Transcription factor jumonji (jmjc) domain-containing protein |
| At3g20840 | TC277111 | Similar to ovule development protein |
| At3g21100 | TC255533 | Rna recognition motif (rrm)-containing protein |
| At3g21400 | TC274701 | Unknown |
| At3g21730 | TC276658 | Dihydroneopterin aldolase family protein |
| At3g22180 | TC266745 | Zinc finger (dhhc type) family protein |
| At3g22220 | TC274342 | Hat dimerisation domain-containing protein |
| At3g22400 | TC262490 | Lipoxygenase |
| At3g23780 | TC268543 | DNA-directed RNA polymerase |
| At3g23900 | TC256433 | Rna recognition motif (rrm)-containing protein |
| At3g24220 | TC257098 | 9-cis-epoxycarotenoid dioxygenase |
| At3g24650 | TC273434 | ABI3 protein |
| At3g25160 | TC274836 | Er lumen protein retaining receptor family protein |
| At3g25870 | TC274449 | Unknown |
| At3g25890 | TC257333 | Ethylene response factor |
| At3g26730 | TC268833 | Zinc finger (C3HC4-type RING finger) family protein |
| At3g26770 | TC255764 | Alcohol dehydrogenase-like protein |
| At3g26790 | TC254343 | Fusca3 |
| At3g27260 | TC266476 | Dna-binding bromodomain-containing protein |
| At3g27660 | TC275195 | Oleosin |
| At3g27870 | TC259551 | Phospholipid-transporting atpase 8 (Aminophospholipid flippase 8) |
| At3g29390 | TC255450 | Hydroxyproline-rich glycoprotein |
| At3g30350 | TC279515 | Unknown |
| At3g30413 | TC278248 | Gypsy-like retrotransposon |
| At3g30436 | TC258958 | Gypsy-like retrotransposon |
| At3g42940 | TC267081 | Unknown |
| At3g44460 | TC254735 | Basic leucine zipper transcription factor |
| At3g44690 | TC258394 | Unknown |
| At3g44830 | TC277330 | Lecithin:cholesterol acyltransferase family protein |
| At3g45130 | TC266003 | Cycloartenol synthase |
| At3g45210 | TC274871 | Unknown |
| At3g46020 | TC275270 | RNA binding protein-like |
| At3g46720 | TC277036 | Glucuronosyl transferase-like protein |
| At3g47220 | TC256796 | Phosphoinositide-specific phospholipase c family protein |
| At3g48580 | TC276359 | Xyloglucan:xyloglucosyl transferase |
| At3g49140 | TC256990 | Unknown |
| At3g49640 | TC268458 | Nitrogen regulation family protein |
| At3g49740 | TC267510 | Pentatricopeptide (ppr) repeat-containing protein |
| At3g50410 | TC256959 | Dof-type zinc finger domain-containing protein |
| At3g50620 | TC276937 | Nodulation protein-related |
| At3g51370 | TC276638 | Protein phosphatase 2C |
| At3g52110 | TC266567 | Unknown |
| At3g52340 | TC274220 | Sucrose-phosphatase |
| At3g53030 | TC264479 | Protein kinase family protein |
| At3g53520 | TC271113 | DTDP-glucose 4-6-dehydratase-like protein |
| At3g54270 | TC256776 | Sucrose-phosphatase 3 (SPP3) |
| At3g54720 | TC264539 | Glutamate carboxypeptidase |
| At3g54860 | TC263524 | Vacuolar protein sorting protein |
| At3g54940 | TC252346 | Cysteine proteinase |
| At3g54970 | TC274670 | Unknown |
| At3g55020 | TC271665 | Rabgap/TBC domain-containing protein |
| At3g55090 | TC268232 | Abc transporter family protein |
| At3g55160 | TC277915 | Unknown |
| At3g56260 | TC266131 | Unknown |
| At3g56270 | TC263812 | Unknown |
| At3g57780 | TC266632 | Unknown |
| At3g58740 | TC254249 | Citrate synthase-like protein |
| At3g58780 | TC256368 | Agamous-like MADS box protein AGL1 (Protein Shatterproof 1) |
| At3g58790 | TC272331 | Glycosyl transferase family 8 protein |
| At3g59850 | TC276255 | Polygalacturonase-like protein |
| At3g60730 | TC275601 | Pectinesterase-like protein |
| At3g61040 | TC256844 | Cytochrome P450 monooxygenase-like protein |
| At3g61340 | TC256774 | F-box family protein |
| At3g61380 | TC276237 | Unknown |
| At3g61640 | TC269184 | Arabinogalactan-protein (AGP20) |
| At3g61690 | TC273636 | Unknown |
| At3g62730 | TC270840 | Desiccation-related protein |
| At3g63040 | TC257442 | Unknown |
| At4g00250 | TC274705 | Dna-binding storekeeper protein-related |
| At4g00540 | TC274911 | Myb family transcription factor |
| At4g00760 | TC256981 | Two-component responsive regulator family protein |
| At4g00790 | TC257825 | Unknown |
| At4g01650 | TC272307 | Unknown |
| At4g01897 | TC259129 | Unknown |
| At4g01970 | TC265816 | Raffinose synthase family protein |
| At4g02000 | TC256985 | Zinc finger protein |
| At4g02030 | TC263409 | Unknown |
| At4g02630 | TC254789 | Serine/threonine-specific protein kinase |
| At4g02740 | TC256802 | F-box family protein |
| At4g04155 | TC269213 | Unknown |
| At4g07960 | TC272947 | Glycosyl transferase family 2 |
| At4g08330 | TC273168 | Unknown |
| At4g09180 | TC274734 | Basic helix-loop-helix (bhlh) family protein |
| At4g09500 | TC274601 | Glycosyltransferase family protein |
| At4g10850 | TC274946 | Nodulin mtn3 family protein |
| At4g12540 | TC267418 | Unknown |
| At4g12870 | TC254854 | Unknown |
| At4g13750 | TC266982 | Unknown |
| At4g14590 | TC266891 | Unknown |
| At4g14600 | TC273938 | Unknown |
| At4g14730 | TC278401 | Transmembrane protein-related |
| At4g14780 | TC274223 | Kinase like protein |
| At4g15020 | TC265088 | Unknown |
| At4g15120 | TC255132 | Vq motif-containing protein |
| At4g15396 | TC266100 | Cytochrome p450-related |
| At4g15417 | TC268052 | Ribonuclease iii family protein |
| At4g16620 | TC257359 | Integral membrane family protein |
| At4g17370 | TC276649 | Oxidoreductase family protein |
| At4g17895 | TC275845 | Ubiquitin-specific protease 20 |
| At4g18910 | TC273199 | Aquaglyceroporin |
| At4g19160 | TC271283 | Unknown |
| At4g19500 | TC254674 | Resistence protein-like |
| At4g21060 | TC277119 | Galactosyltransferase family protein |
| At4g21380 | TC255785 | S-receptor kinase ARK3 precursor |
| At4g22100 | TC259055 | Glucosidase like protein |
| At4g22120 | TC258222 | Early-responsive to dehydration protein-related |
| At4g22290 | TC254214 | Ubiquitin carboxyl-terminal hydrolase family protein |
| At4g22390 | TC257645 | F-box family protein-related |
| At4g22560 | TC275720 | Unknown |
| At4g22820 | TC271149 | Zinc finger (an1-like) family protei |
| At4g22850 | TC274318 | Unknown |
| At4g23520 | TC266928 | Cysteine proteinase |
| At4g25140 | TC274793 | Oleosin |
| At4g25750 | TC266771 | Abc transporter family protein |
| At4g26400 | TC262098 | Zinc finger (c3hc4-type ring finger) family protein |
| At4g26420 | TC265484 | S-adenosyl-L-methionine:salicylic acid carboxyl methyltransferase-like protein |
| At4g27040 | TC253797 | SNF8 like protein |
| At4g27150 | TC261110 | 2S seed storage protein 2 precursor |
| At4g27420 | TC258290 | Abc transporter family protein |
| At4g27460 | TC265034 | Cbs domain-containing protein |
| At4g28520 | TC270748 | 12S cruciferin seed storage protein (CRU3) |
| At4g28530 | TC254906 | CUC2-like protein |
| At4g28760 | TC278810 | Unknown |
| At4g28950 | TC265683 | Rac GTP binding protein Arac7 |
| At4g29070 | TC265576 | Unknown |
| At4g29240 | TC272940 | Extensin-like protein |
| At4g32000 | TC268678 | Serine/threonine protein kinase like protein |
| At4g32295 | TC275317 | Unknown |
| At4g32700 | TC257335 | Dna-directed dna polymerase family protein |
| At4g32940 | TC268598 | Vacuolar processing enzyme gamma |
| At4g33180 | TC267141 | Hydrolase, alpha/beta fold family protein |
| At4g33280 | TC266997 | Auxin response factor 36 |
| At4g33500 | TC273896 | BTH-induced protein phosphatase 1 |
| At4g33800 | TC275350 | Unknown |
| At4g33820 | TC257012 | Glycosyl hydrolase family 10 protein |
| At4g33980 | TC264760 | Unknown |
| At4g35335 | TC255677 | UDP-galactose transporter-like protein |
| At4g35410 | TC263874 | Clathrin adaptor complex small chain family protein |
| At4g35500 | TC264619 | Protein kinase family protein |
| At4g36190 | TC270120 | Serine carboxypeptidase s28 |
| At4g36280 | TC275650 | Atpase-like domain-containing protein |
| At4g36630 | TC273819 | Unknown |
| At4g36700 | TC251621 | Globulin-like protein |
| At4g36910 | TC264005 | Cbs domain-containing protein |
| At4g36930 | TC255215 | Spatula |
| At4g37050 | TC263764 | Patatin |
| At4g38560 | TC265789 | Phospholipase like protein |
| At4g38570 | TC268772 | CDP-diacylglycerol--inositol 3-phosphatidyltransferase |
| At4g39390 | TC263521 | Glucose-6-phosphate/phosphate-translocator precursor |
| At4g39510 | TC264187 | Cytochrome P450-like protein |
| At4g39930 | TC275293 | Unknown |
| At5g01470 | TC266425 | Unknown |
| At5g01670 | TC258251 | Aldose reductase-like protein |
| At5g01780 | TC276465 | Oxidoreductase |
| At5g03800 | TC274332 | Limonene cyclase |
| At5g03860 | TC271861 | Malate synthase |
| At5g04010 | TC277811 | Unknown |
| At5g04370 | TC259603 | S-adenosyl-l-methionine:carboxyl methyltransferase family protein |
| At5g04620 | TC265939 | Aminotransferase class i and ii family protein |
| At5g05070 | TC276648 | Zinc finger (dhhc type) family protein |
| At5g06830 | TC257469 | CDK5RAP3-like protein |
| At5g07190 | TC271632 | Embryo-specific protein 3 |
| At5g07260 | TC255657 | Homeobox protein-related |
| At5g07280 | TC276451 | Leucine-rich repeat protein kinase |
| At5g07500 | TC256536 | Zinc finger transcription factor |
| At5g07890 | TC257058 | Myosin heavy chain-related |
| At5g07990 | TC271200 | Flavonoid 3'-monooxygenase |
| At5g08170 | TC272770 | Peptidyl-arginine deiminase-like protein |
| At5g08430 | TC276564 | Swib complex baf60b domain-containing protein |
| At5g08460 | TC266506 | GDSL-motif lipase/acylhydrolase-like protein |
| At5g08480 | TC276819 | Vq motif-containing protein |
| At5g08535 | TC278158 | D111/G-patch domain-containing protein |
| At5g09640 | TC253332 | Serine carboxypeptidase |
| At5g09840 | TC277416 | Unknown |
| At5g10460 | TC255016 | Haloacid dehalogenase-like hydrolase family protein |
| At5g10700 | TC267157 | Unknown |
| At5g11110 | TC264890 | Sucrose-phosphate synthase |
| At5g11170 | TC268407 | DEAD/DEAH box helicase, putative (RH15) |
| At5g11240 | TC254871 | Transducin |
| At5g11310 | TC266147 | Pentatricopeptide (PPR) repeat-containing protein-like |
| At5g11320 | TC276472 | Flavin-containing monooxygenase family protein |
| At5g11650 | TC265511 | Hydrolase, alpha/beta fold family protein |
| At5g11840 | TC255973 | Unknown |
| At5g13480 | TC278489 | Wd-40 repeat family protein |
| At5g13520 | TC263893 | Leukotriene-A4 hydrolase-like protein |
| At5g13570 | TC275182 | Mrna-decapping enzyme |
| At5g13690 | TC254617 | Alpha-N-acetylglucosaminidase |
| At5g13770 | TC267444 | Pentatricopeptide (ppr) repeat-containing protein |
| At5g13790 | TC276123 | Agamous-like MADS box protein AGL15 |
| At5g15020 | TC276581 | Paired amphipathic helix repeat-containing protein |
| At5g15440 | TC255045 | Circadian clock coupling factor-related |
| At5g15470 | TC263803 | Glycosyl transferase family 8 protein |
| At5g15710 | TC274215 | F-box family protein |
| At5g15940 | TC268006 | Short-chain dehydrogenase/reductase |
| At5g16070 | TC252747 | Chaperonin |
| At5g16310 | TC254090 | Ubiquitin carboxyl-terminal hydrolase family 1 protein |
| At5g17040 | TC266142 | UDP glucose:flavonoid 3-o-glucosyltransferase |
| At5g18390 | TC269516 | Pentatricopeptide (ppr) repeat-containing protein |
| At5g18420 | TC272968 | Unknown |
| At5g18840 | TC265257 | Sugar transporter-like protein |
| At5g19730 | TC276026 | Pectinesterase family protein |
| At5g19850 | TC275884 | Hydrolase, alpha/beta fold family protein |
| At5g20040 | TC262325 | Trna isopentenyltransferase 9 |
| At5g20420 | TC268871 | Snf2 domain-containing protein |
| At5g22030 | TC273376 | Ubiquitin-specific protease-like protein |
| At5g22470 | TC254203 | Poly (ADP-ribose) polymerase family protein |
| At5g22500 | TC261409 | Acyl coa reductase |
| At5g22730 | TC256230 | F-box family protein |
| At5g22810 | TC257381 | GDSL-motif lipase/hydrolase-like protein |
| At5g23520 | TC273156 | Unknown |
| At5g24470 | TC254105 | Pseudo-response regulator 5 |
| At5g24950 | TC268102 | Cytochrome P450 71A15 |
| At5g26120 | TC273481 | Glycosyl hydrolase family protein 51 |
| At5g26240 | TC265629 | Chloride channel protein CLC-d |
| At5g26760 | TC266335 | Unknown |
| At5g26850 | TC262492 | Unknown |
| At5g27360 | TC275742 | Sugar-porter family protein 2 (SFP2) |
| At5g27610 | TC257308 | Always early 1 protein |
| At5g27950 | TC274870 | BY-2 kinesin-like protein 5 |
| At5g28910 | TC268406 | Unknown |
| At5g35450 | TC264568 | Disease resistance protein |
| At5g35790 | TC273100 | Plastidic glucose-6-phosphate dehydrogenase |
| At5g37580 | TC267424 | Unknown |
| At5g37590 | TC278438 | Kinesin light chain-related protein |
| At5g38110 | TC273956 | Anti-silencing protein-like |
| At5g38160 | TC255960 | Lipid transfer like protein |
| At5g38830 | TC276343 | Cysteine-trna ligase |
| At5g39130 | TC273382 | Germin-like protein subfamily 1 member 16 precursor |
| At5g40420 | TC251445 | Oleosin |
| At5g41140 | TC274181 | Unknown |
| At5g41150 | TC274132 | Repair endonuclease |
| At5g41330 | TC256624 | Potassium channel tetramerisation domain-containing protein |
| At5g41580 | TC256654 | Transcription factor-like protein |
| At5g42320 | TC257350 | Zinc carboxypeptidase family protein |
| At5g42670 | TC258664 | Agenet domain-containing protein |
| At5g42800 | TC271692 | Dihydroflavonol 4-reductase |
| At5g43020 | TC256511 | Leucine-rich repeat transmembrane protein kinase |
| At5g44050 | TC277019 | Mate efflux family protein |
| At5g44310 | TC254966 | Late embryogenesis abundant protein-like |
| At5g45690 | TC273255 | Unknown |
| At5g45760 | TC256088 | Transducin family protein |
| At5g45770 | TC265475 | Leucine-rich repeat family protein |
| At5g45830 | TC267134 | Similarity to tumor-related protein |
| At5g45850 | TC258406 | Unknown |
| At5g46400 | TC258157 | Unknown |
| At5g46460 | TC277932 | Pentatricopeptide (ppr) repeat-containing protein |
| At5g46540 | TC268647 | Abc transporter family protein |
| At5g46870 | TC257030 | Rna recognition motif (rrm)-containing protein |
| At5g47150 | TC277548 | Similarity to SET-domain protein |
| At5g47670 | TC256401 | Leafy cotyledon 1-like L1L protein |
| At5g47720 | TC271603 | Acetoacyl-coa-thiolase |
| At5g47800 | TC274529 | Phototropic-responsive nph3 family protein |
| At5g48100 | TC251742 | Laccase |
| At5g48360 | TC273570 | Formin homology 2 domain-containing protein |
| At5g48485 | TC273176 | Lipid transfer protein (ltp) family protein |
| At5g49190 | TC263845 | Sucrose synthase |
| At5g49950 | TC264522 | Embryogenesis-associated protein-related |
| At5g50260 | TC263997 | Cysteine endopeptidase |
| At5g50480 | TC266431 | Transcription factor Hap5a-like |
| At5g50650 | TC265297 | Protein transport protein SEC12p-like |
| At5g50700 | TC270936 | 11-beta-hydroxysteroid dehydrogenase-like |
| At5g50750 | TC263858 | Reversibly glycosylated polypeptide RGP-4 |
| At5g50770 | TC278807 | Short-chain dehydrogenase/reductase (SDR) family protein |
| At5g50790 | TC254997 | Mtn3-like protein |
| At5g51500 | TC267997 | Pectinesterase |
| At5g51690 | TC263304 | 1-aminocyclopropane-1-carboxylate synthase (ACC synthase) |
| At5g51810 | TC274586 | Gibberellin 20-oxidase |
| At5g51850 | TC276893 | Unknown |
| At5g51870 | TC268610 | Mads-box protein (agl71) |
| At5g52330 | TC258063 | Meprin and traf homology domain-containing protein |
| At5g52860 | TC266648 | ABC transporter-like protein |
| At5g53280 | TC275202 | Unknown |
| At5g53440 | TC275001 | Unknown |
| At5g53750 | TC267570 | Cbs domain-containing protein |
| At5g54740 | TC261462 | Lipid transfer protein (ltp) family protein |
| At5g55180 | TC257483 | Beta-1,3-glucanase-like protein |
| At5g55240 | TC254176 | Caleosin-related family protein |
| At5g55410 | TC255953 | Lipid transfer protein (ltp) family protein |
| At5g56300 | TC276993 | S-adenosyl-L-methionine:salicylic acid carboxyl methyltransferase-like protein |
| At5g56370 | TC266189 | F-box family protein |
| At5g56700 | TC278829 | F-box family protein |
| At5g57140 | TC264136 | Calcineurin-like phosphoesterase family protein |
| At5g57260 | TC275691 | Cytochrome P450 |
| At5g57390 | TC274827 | AP2/EREBP transcription factor |
| At5g57700 | TC255398 | BNR/Asp-box repeat family protein |
| At5g57790 | TC268409 | Unknown |
| At5g58080 | TC277362 | Two-component responsive regulator family protein |
| At5g59170 | TC272223 | Cell wall protein precursor, extensin |
| At5g59190 | TC277337 | Subtilase family protein |
| At5g59300 | TC266325 | Ubiquitin-conjugating enzyme 7 (ubc7), e2 |
| At5g59350 | TC273984 | Unknown |
| At5g59590 | TC274208 | Udp-glucoronosyl/udp-glucosyl transferase family protein |
| At5g59845 | TC264606 | Gibberellin-regulated family protein |
| At5g60610 | TC276067 | F-box family protein |
| At5g60760 | TC255178 | 2-phosphoglycerate kinase-related |
| At5g61150 | TC263854 | Vernalization independence 4 |
| At5g61390 | TC257077 | Exonuclease-like protein |
| At5g62170 | TC274808 | Unknown |
| At5g62490 | TC254087 | Athva22b |
| At5g62800 | TC268787 | Seven in absentia (SINA) family protein |
| At5g62840 | TC265463 | Phosphoglycerate/bisphosphoglycerate mutase family protein |
| At5g63000 | TC274778 | Unknown |
| At5g63080 | TC266876 | Transcription factor jumonji (jmjc) domain-containing protein |
| At5g63120 | TC254559 | ATP-dependent RNA helicase-like protein |
| At5g63160 | TC277582 | Speckle-type POZ protein-related |
| At5g63610 | TC263695 | Cyclin-dependent kinase cdc2mse |
| At5g63760 | TC254738 | ARIADNE-like protein ARI15 |
| At5g64200 | TC264554 | Arginine/serine-rich splicing factor SC35 |
| At5g64900 | TC264263 | Unknown |
| At5g65165 | TC277079 | Succinate dehydrogenase, iron-sulphur subunit, mitochondrial |
| At5g66180 | TC265603 | Similar to nol1/nop2/sun family protein |
| At5g67240 | TC255283 | Exonuclease |
| AtMg00180 | TC278282 | Ccb452 cytochrome c biogenesis orf452 |
| AtMg00270 | TC259657 | NADH-ubiquinone oxidoreductase chain 6 |
| AtMg00520 | TC267036 | Maturase |
| Chloroplast genome | TC262117 | Photosystem I assembly protein Ycf3 |
| Chloroplast genome | TC258979 | Unknown |
| AA651576 | TC260948 | Arabidopsis thaliana 18S rrna gene |
| AC004557 | TC253861 | Unknown |
| AC006954 | TC269089 | Retroelement |
| AC007534 | TC263707 | Retroelement |
| AF074021 | TC258129 | Transposon |
| AY072203 | TC274611 | Transposon |
| AY088691 | TC258748 | Unknown |
| BE520573 | TC259174 | Unknown |
| BE520635 | TC258019 | 12S seed storage protein |
| BE520744 | TC268594 | Leupaxin |
| BE520891 | TC278755 | Unknown |
| BE520985 | TC259388 | Unknown |
| BE521055 | TC257247 | Unknown |
| BE521057 | TC259044 | Unknown |
| BE521384 | TC268730 | Unknown |
| BE521533 | TC268737 | Unknown |
| BE521633 | TC278198 | Unknown |
| BE522534 | TC278435 | Unknown |
| BE523353 | TC259401 | Unknown |
| BE524156 | TC278329 | Unknown |
| BE525582 | TC258944 | Unknown |
| BE523013 | TC259196 | Unknown |
| BE523713 | TC278028 | Unknown |
| BE528808 | TC268646 | Unknown |
| BE530021 | TC277499 | Unknown |
| BE530849 | TC268861 | Unknown |
